# Association between Development Assistance for Health and Disease Burden: A Longitudinal Analysis on Official Development Assistance for HIV/AIDS, Tuberculosis, and Malaria in 2005–2017

**DOI:** 10.3390/ijerph192114091

**Published:** 2022-10-28

**Authors:** Sumin Kim, Ermias Tadesse, Yan Jin, Seungman Cha

**Affiliations:** 1 Department of Global Development and Entrepreneurship, Graduate School of Global Development and Entrepreneurship, Handong Global University, Pohang 37554, Korea; 2 Department of Clinical Research Design and Evaluation, Samsung Advanced Institute for Health Sciences & Technology (SAIHST), Sungkyunkwan University, Seoul 06355, Korea; 3 Department of Pediatrics, Samsung Medical Center, Sungkyunkwan University School of Medicine, Seoul 06355, Korea; 4 Department of Human Ecology and Technology, Graduate School of Advanced Convergence, Handong Global University, Pohang 37554, Korea; 5 Department of Microbiology, Dongguk University College of Medicine, Gyeongju 38066, Korea

**Keywords:** development assistance for health, HIV/AIDS, TB and malaria, disease burden

## Abstract

From the early stage of the millennium development goals campaign, HIV/AIDS, tuberculosis and malaria have received huge aid funds. With the datasets published by the Institute for Health Metrics and Evaluation, Organization for Economic Cooperation and Developments, and World Health Organization from 2005 to 2017, we analyzed the association between the total DAH or DAH per capita and the disease burden. We measured the total DAH or DAH per capita as the dependent variable, with six independent variables of disease burden for Disability Adjusted Life Year (DALY), number of infected people, number of deaths, prevalence, incidence, and mortality rate. For the trend in ODA targeting, the likelihood ratio test of the fixed effects models was used to assess any existence of slope changes in linear regression across the years. The total amount of DAH and DAH per capita was found positively related with every aspect of disease burden, with the regression coefficients increasing during 2005–2017. For instance, the slope of association between the DAH per capita and the disease burden of malaria became steeper over time (likelihood ratio, χ^2^ = 26.14, *p* < 0.001). Although the selection criteria for the recipient country have been controversial, ODA targeting has been performed based on disease burden in this research.

## 1. Background

Universal health care (UHC) is the ultimate goal of the global health community [1]. However, the COVID-19 outbreak has been a stumbling block for achieving UHC, putting more pressure on healthcare and financing systems in many developing countries [2]. In this challenging environment, it is more crucial for the global health community to make informed decisions regarding the allocation of aid for health in developing countries [3].

HIV/AIDS, tuberculosis (TB), and malaria are the main public health concerns in many developing countries, and fighting these diseases is still one of the top priorities on the global health agenda [3,4]. In fact, HIV/AIDS, TB, and malaria claimed 26.4 million lives between 2013 and 2017 in developing countries [5]. In response to this massive disease burden, there have been tremendous efforts taken to tackle these diseases across the world [6]. Accordingly, the issues of the quality and effectiveness of aid have been frequently raised, and the importance of appropriate monitoring and the evaluation of aid projects has recently been further emphasized [7,8]. However, Official Development Assistance (ODA) targeting has received relatively less attention [3]. It has not been well documented whether development assistance for health has been allocated in proportion to disease burden [6,7,8].

ODA covers one-fifth of the health expenditures in least developed countries (LDC) [5]. Half of the total health ODA each year goes to sub-Saharan Africa, where they have the highest disease burden [9]. When aid volatility is high, many developing countries relying on aid suffer unpredictability in their resource availability [4]. Development assistance for health (DAH) is defined as financial and in-kind donations made to low- and middle-income countries in order to maintain or improve health through development institutions [10]. DAH has played a significant role in combating HIV/AIDS, TB, and malaria [6]. Although effective interventions do exist for tackling these diseases, many developing countries have been encountering barriers in expanding health services, mainly due to the lack of resources [11]. The vulnerability to DAH reductions in low- and middle-income countries has been frequently reported [12,13]. From 2000 to 2009, DAH were increased by 11.3% per year, including HIV/AIDS, TB, and malaria, but have remained almost unchanged since 2010 [14]. Indeed, volatility and unpredictability make it difficult for recipient countries to develop adequate health strategies to tackle key public health issues [15].

It has been reported that primarily allocating DAH by the disease burden reduces volatility and unpredictability [16]. This points to the importance of investigating whether aid for healthcare has been properly allocated by the disease burden in the recipient countries.

In recent years, some studies have been tracking ODA investment in HIV/AIDS, TB, and malaria [17,18,19,20,21,22]. However, most existing investigations have focused on exploring the effects of DAH on health improvement, rather than the adequacy of DAH allocation. Hsiao and colleagues investigated whether DAH targeted specifically to HIV, TB, and malaria was associated with changes in malaria, HIV, and TB mortality, respectively [21]. Similarly, Yan and colleagues assessed the health impact of the Global Fund to Fight AIDS, Tuberculosis, and Malaria (Global Fund) [22]. This study considered the relationship between DAH and disease burden, and was mainly focused on the global level, not the country level [23]. A few studies have assessed the extent to which donors have targeted development assistance to countries with the highest rates of maternal and under-5 mortality [24,25]. We aimed to extend the assessment to HIV/AIDS, TB, and malaria by focusing on the extent to which donors targeted DAH to countries with the greatest burden.

In understanding to what extent DAH is being allocated in relation to the disease burden of HIV/AIDS, TB, and malaria, a longitudinal analysis of the time trend may provide some evidence for the global health community in order to help them make informed choices for effective resource allocation.

Therefore, this study investigated whether donors are allocating their ODA in proportion to the disease burden by assessing associations between aid allocation and disease burden. This study also investigated time trends of ODA targeting in 2005–2017. For disease burden, we used a range of variables such as DALY, incidence, number of deaths, and prevalence of each disease, and assessed the associations between DAH and disease burden at both an individual and country level.

## 2. Methods

### 2.1. Target Countries

We selected 92, 87, and 68 endemic countries for the HIV/AIDS, TB, and malaria study, respectively [26]. The full list of the countries is described in Supplementary Materials.

### 2.2. Data Source

We collected DAH data from the Institute for Health Metrics and Evaluation (IHME, Seattle, WA, USA) for the period of 2005–2017 [27]. Multiple datasets are available to track DAH, and the strengths and weaknesses of each dataset have been described elsewhere [28]. The limitations of the Organization for Economic Cooperation and Developments (OECD) Development Assistance Committee (DAC) datasets are that they are insufficiently comprehensive, do not account for all resource contributions from an individual donor country, and only capture limited data from some global health initiatives, non-DAC bilateral donors, NGOs, or foundations, with a few exceptions [29]. The IHME has developed its own DAH databases specifically to track health projects. The IHME complements the OECD databases with additional data collected from reports, financial statements, online databases, and tax filings, as well as other sources of information. It uses a broader definition of aid, including both ODA and non-ODA flows, aid provided through private donors such as NGOs and foundations, and loans from the International Bank for Reconstruction.

We used the global burden of disease (GBD) reports for disability adjustment life year (DALY), annual number of deaths from HIV/AIDS, TB, and malaria [5,30]. For the number of cases of TB and malaria, and the number of infected people with HIV/AIDs, the incidence of HIV/AIDS (per 1000 uninfected population ages 15–49), malaria incidence rate (per 1000 population at risk), and incidence of TB (per 100,000 population per year), we used the data published by the world health organization (WHO) [5]. For GNI per capita and population, the World Bank Group reports were used [31,32].

### 2.3. Analysis

We examined the total amount of disbursement of DAH for HIV/AIDS, TB, and malaria and the annual changes of DAH or DAH per capita from 2005 to 2017. For examining the association between DAH and disease burden each year, we ran an ordinary least square regression analysis, controlling for GNI per capita. For the dependent variable, we used the total DAH or DAH per capita received per year. We used disease burden as the independent variable.

For the disease burden, we used DALYs, the number of infected people, and the number of deaths when the dependent variable was the total DAH. On the other hand, when we measured the DAH per capita, and we used incidence, prevalence, and mortality as the independent variables. We made a log transformation for both the dependent and independent variables. We also examined whether the slope of the linear regression between DAH and disease burden was significantly changed over time. Data from 2005 to 2017 were pooled to test for a difference in the slope after setting two fixed effects models. We allowed the slope to vary year by year in one model, and assumed the same slope in another model over time, and then ran the likelihood ratio test between the two models. We used the variance inflation factor (VIF) to test for multicollinearity and the Durbin–Watson statistic to test for autocorrelation. We used STATA (version 16.0) for the analyses. We used STATA (version 16.0) for the analyses.

## 3. Results

**Figure 1:**
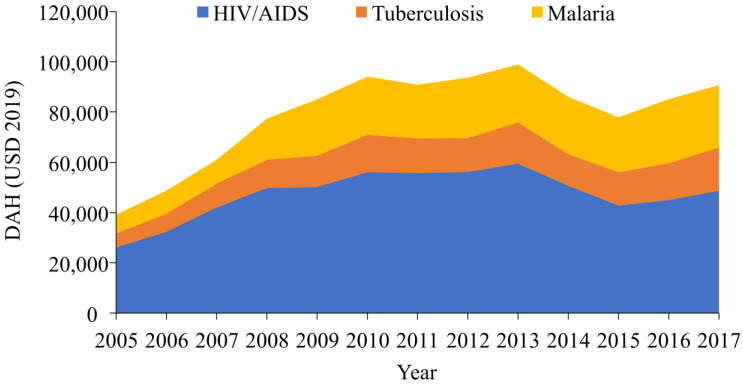
Stacked area chart, development assistance for health to HIV/AIDS, TB, and malaria, 2005–2017 (unit of *y*-axis: millions of 2019 USD, DAH = development assistance for health).

Table 1 presents the total amount of DAH, DAH per capita, and the annual changes in 2005–2017. The amount of the total DAH increased since 2005, but showed a slight decrease around 2014–2016. There were substantial increases in DAH for HIV/AIDS and TB in 2006–2007, while the annual change of DAH for malaria peaked in 2008–2009 (Table 1 and Figure 1). 

**Figure 2:**
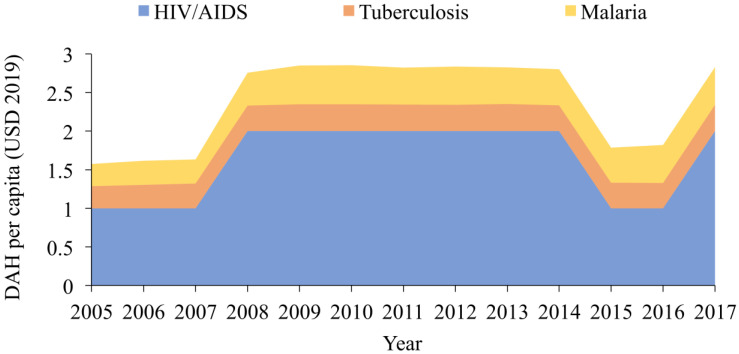
Stacked area chart, development assistance for health per capita to HIV/AIDS, TB and malaria, 2005–2017 (unit of *y*-axis: 10 million of 2019 USD, DAH = development assistance for health).

Figure 2 demonstrates the trend of DAH per capita for each disease. DAH per capita for HIV/AIDS, TB, and malaria showed a rapid increase in 2007 and remained almost constant until 2014, then abruptly decreased from 2014. HIV/AIDS received the highest amount of DAH in every single year in terms of both the total amount of DAH and DAH per capita (Figure 1 and Figure 2).

**Figure 3:**
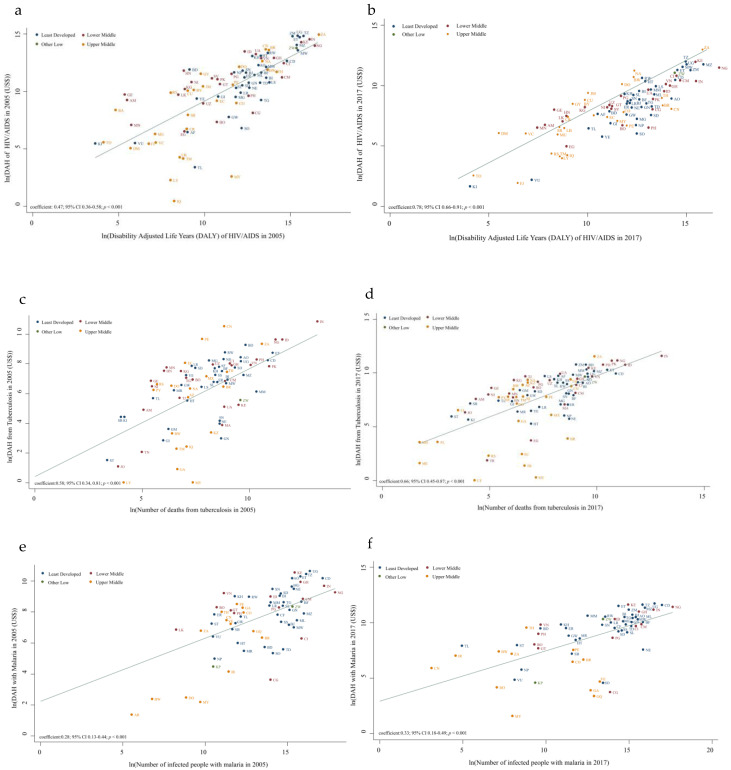
Associations between development assistance for health and disease burden (*x*-axis: disease burden, log transformation, *y*-axis: DAH (USD), log transformation, income group classification by World Bank are colored). (**a**) DAH and Disability Adjusted Life Years (DALY) of HIV/AIDS in 2005. (**b**) DAH and Disability Adjusted Life Years (DALY) of HIV/AIDS in 2017. (**c**) DAH and number of deaths from tuberculosis (TB) in 2005. (**d**) DAH and number of deaths from TB in 2017. (**e**) DAH and number of infected people with malaria in 2005. (**f**) DAH and number of infected people with malaria in 2017. Afghanistan (AF), Albania (AL), Algeria (DZ), Angola (AO), Armenia (AM), Azerbaijan (AZ), Argentina (AR), Bangladesh (BD), Belarus (BY), Benin (BJ), Bolivia (BO), Bosnia and Herzegovina (BA), Botswana (BW), Brazil (BR), Burkina Faso (BF), Burundi (BI), Cambodia (KH), Cameroon (CM), Central African Republic (CF), Chad (TD), China (CN), Colombia (CO), Congo (CG), Costa Rica (CR), Cote d’Ivoire (CI), Cuba (CU), Democratic People’s Republic of Korea (KP), Democratic Republic of the Congo (CD), Djibouti (GI), Dominica (DM), Dominican Republic (DO), Ecuador (EC), Egypt (EG), El Salvador (SV), Equatorial Guinea (GQ), Eritrea (ER), Ethiopia (ET), Fiji (FJ), Gabon (GA), Gambia (GM), Georgia (GE), Ghana (GH), Guatemala (GT), Guinea (GN), Guinea-Bissau (GW), Guyana (GY), Haiti (HT), Honduras (HN), India (IN), Indonesia (ID), Iran (IR), Iraq (IQ), Jordan (JO), Jamaica (JM), Kazakhstan (KZ), Kenya (KE), Kiribati (KI), Kyrgyzstan (KG), Lebanon (LB), Lesotho (LS), Liberia (LR), Libya (LY), Madagascar (MG), Malawi (MW), Malaysia (MY), Mali (ML), Marshall Islands (MH), Mauritania (MR), Mexico (MX), Mauritius (MU), Mongolia (MN), Montenegro (ME), Morocco (MA), Mozambique (MZ), Myanmar (MM), Namibia (NA), Nepal (NP), Nicaragua (NI), Niger (NE), Nigeria (NG), Pakistan (PK), Papua New Guinea (PG), Paraguay (PY), Peru (PE), Philippines (PH), Rwanda (RW), Saint Vincent and the Grenadines (VC), Sao Tome and Principe (ST), Senegal (SN), Serbia (RS), Sierra Leone (SL), Solomon Islands (SB), Somalia (SO), South Africa (ZA), Sri Lanka (LK), South Sudan (SS), Sudan (SD), Suriname (SR), Tanzania (TZ), Tajikistan (TJ), Thailand (TH), Timor-Leste (TL), Togo (TG), Tonga (TO), Tunisia (TN), Turkey (TR), Turkmenistan (TM), Uganda (UG), Ukraine (UA), Uzbekistan (UZ), Vanuatu (VU), Venezuela (VE), Vietnam (VN), Yemen (YE), Zambia (ZM), Zimbabwe (ZW).

Table 2 and Figure 3 present the results of the associational analysis between the DAH amount and disease burden. All of the results from 2005 throughout 2017 are described in the Supplementary Materials. The total amount of DAH and DAH per capita were associated with disease burden, with a very few exceptions. For instance, as DALY increased, the total DAH to HIV/AIDS increased (coefficient: 0.79, 95% CI 0.66–0.9, *p* < 0.001) in 2017. Similarly, DAH per capita to TB increased per incidence increase (coefficient: 0.21, 95% CI 0.12–0.31, *p* < 0.001) in 2017. For malaria, the DAH per capita increased per mortality increase (coefficient: 0.04, 95% CI −0.05–0.12, *p* < 0.001) in 2017. Overall, the coefficient increased over time during the study period. The analysis results for the association for every single year from 2005 throughout 2017 are presented in the Supplementary Materials. The results of the multicollinearity indicated a very low correlation between the independent variables (most of the VIFs were less than 2, a few VIFs were between 2–2.5).

Table 3 presents the results of the likelihood ratio test of the two fixed effects models. There were significant changes in slopes across years with a few exceptions. For instance, the likelihood ratio of the two models of the total DAH and DALY of HIV/AIDS was χ^2^ = 47.26 (*p* < 0.001). This suggests that the association between the total DAH and disease burden became stronger over time. The same holds true for malaria and TB. The slope of regression between the DAHs per capita and disease burden became steeper over time (likelihood ratio malaria to prevalence, χ^2^ = 56.69, *p* < 0.001). The results of the Durbin-Watson test showed a very low level of autocorrelation (all were closely around 2)**.**

**Figure 4:**
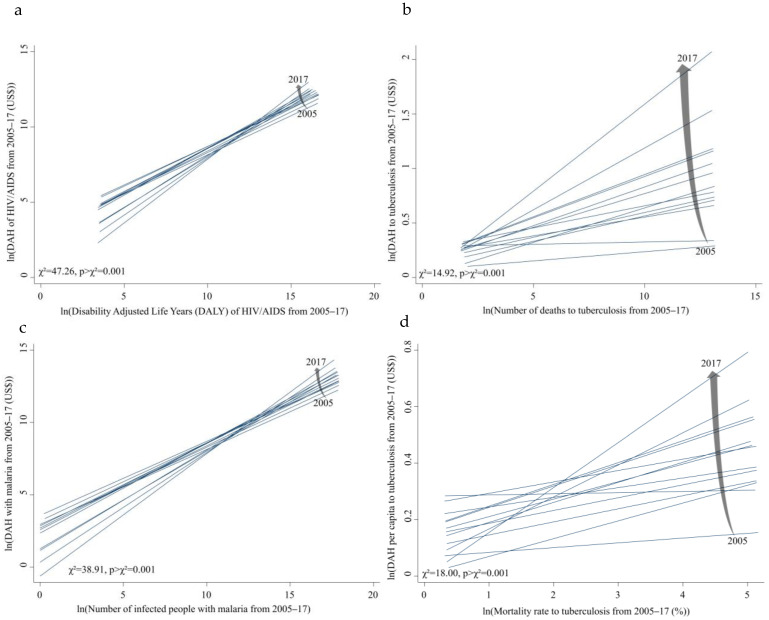
Slope of the linear fitted regression line, 2005–2017, (*x*-axis: disease burden, log transformation, *y*-axis: DAH or DAH per capita (US$), log transformation). (**a**) DAH and Disability Adjusted Life Years (DALY) of HIV/AIDS from 2005 throughout 2017. (**b**) DAH and number of deaths to TB from 2005 throughout 2017. (**c**) DAH and number of infected people with malaria from 2005 throughout 2017. (**d**) DAH per capita and mortality rate to TB from 2005 throughout 2017.

Figure 4 illustrates the slope changes over time. The association became stronger between DAH per capita to TB burden. The results demonstrate that resources under the burden of disease are becoming properly supported over time.

## 4. Discussion

Our study revealed that the amounts of DAH and DAH per capita are correlated with the burden of three diseases. This pattern appears to have increased in strength from 2005 to 2017 when the global health community was considering DAH regarding HIV/AIDS, TB, and malaria in relation to the disease burden of each recipient country. This suggests that there have been continued efforts by global development partners including bilateral and multilateral donors, NGOs, and foundations to make appropriate targeting when selecting their partner countries in order to reduce the disease burden of HIV/AIDS, TB, and malaria. This study also found that the international community is moving in a good direction [33,34,35].

There have been some concerns regarding aid allocation. One of the concerns is that the priority of the aid is set by the interests of the donor country and its political background [33,34,35]. Similarly, there has been a criticism that aid has been allocated with no clear criteria, such as disease burden, or without any fair or transparent processes [36,37].

We acknowledge that it is difficult to interpret whether the mere expansion of aid could lead to improved health outcomes, as governance, health systems, and many other factors could affect the complex relationship between DAH and the improvement in health [38]. Another concern over the aid allocation process is that it seems to rely on the donor’s own unilateral criteria and procedures, without involving partner countries [20]. For a similar reason, we had difficulty obtaining details regarding the aid allocation criteria and processes. These concerns have sometimes led to a suspicion that there might be some hidden political intentions from donor countries or the international communities behind ODA [39]. For this reason, there is a pressing need to increase the transparency of the aid allocation process and criteria, and to develop more reliable and relevant indicators for adequate resource allocation of ODA.

One of these indicators is GNI, which has been used to evaluate the need for aid in recipient countries [34,40]. However, this method has some weaknesses. One of them is that if the DAH allocation for HIV/AIDS, TB, and malaria depends solely on the GNI, some countries with a relatively higher GNI and high disease burden might be excluded [41]. Another is that aid in the countries with a lower GNI with a relatively smaller disease burden might bring another important issue regarding effectiveness. The finding of this study, however, contradicts these arguments by showing that the DAH for the three communicable diseases is strongly associated with the needs of the recipient country in terms of the disease burden. We categorized DAH into total DAH and DAH per capita. The total DAH is not suitable for understanding the average amount of aid provided to the individuals of the recipient country [42]. This study also shows the time trend over a 13 year period with respect to the associations between aid allocation and disease burden. For all three communicable diseases, the associations between disease burden and aid were found to increase over time. All in all, our results show that ODA was adequately disbursed in proportion to disease burden, and in that aid targeting steadily improved over time. The advent of the global financial crisis in 2007–2008 has raised concerns about development assistance in recipient countries [40]. This crisis eventually led to a decrease in aid, which indicates that aid in the health sector becomes highly dependent on and vulnerable to the changing environment.

One silver lining is that DAH is growing rapidly. The total ODA increased by 77% from 2000 to 2014, and the DAH increased by 332% in same period [9]. However, this is still far from the demand required for achieving the SDGs [6,40]. In 2015, the development financing summit was held in Addis Ababa, but there were no new commitments [43]. It has been long overdue to reinvigorate the urgent demands of the continued support from the global community in order to accelerate the progress in SDGs. To this end, cooperation between bilateral and multilateral donors as well as partner countries is crucial [44,45].

Apart from disease burden, there could be a combination of criteria used when selecting recipient countries for HIV/AIDS, TB, and malaria, such as governance, transparency, and management capacity. We could not control for these potential confounding variables in our analyses due to the lack of relevant data, which is a limitation of this study. Further research is warranted in order to understand how much investment is needed to achieve the health sector’s goals of tackling HIV/AIDS, TB, and malaria. There is still lack of information regarding the country-specific business plans for controlling HIV/AIDS, TB, and malaria [38,39]. For a follow-up study to investigate the associations between DAH allocation and disease burden, other indicators such as QALY could be selected. More research is needed to identify adequate strategies to support the countries of substantial disease burden, but who have a with a low transparency, low political stability, and weak governance and health systems.

Dieleman and colleagues pointed out the lack of alignment between DAH and disease burden at the global level, highlighting a mismatch between DAH and the burden of non-communicable diseases [23]. Our study neither explored the alignment of DAH and disease burden at the global level, nor investigated whether DAH was allocated depending on the disease burden within a country. Therefore, this study does not provide any information regarding the adequacy of DAH for HIV/AIDS, TB, and malaria in comparison with other disease burdens at a global or national level, although this issue also warrants future research.

There have been many arguments about how donors select target countries for aid in the health sector as well as the criteria being used [33,34,35]. Our study may not provide final conclusions regarding these existing controversies. Further research is still needed to determine what kind of criteria and procedures should be used for allocating aid to the health sector. It is the responsibility of the entire international health community to track whether the resources invested in the health sector are being used in the right place.

For allocating funds globally, there is still no national program model for HIV/AIDS, TB, and malaria for providing preferential assistance to the most at-risk population groups [46]. We strongly urge the global health community to develop a platform to share the allocation criteria of DAH and to provide relevant information on budgeting, evaluating health expenditures, and setting priorities. Health expenditure has increased significantly over the past two decades. At the beginning of this study, the outbreak of COVID-19 forced healthcare systems around the world to invest huge amounts of financial resources into fighting this pandemic. Even under this circumstance, every effort to achieve UHC, the ultimate goal of the SDGs, must be undertaken. Our research may give insights into how the global health community accelerates the achievement of SDG3.

## 5. Conclusions

We conducted a longitudinal analysis of the associations regarding the ODA amount with disease burden, using datasets published by the WHO, IHME, and OECD. We used comprehensive indicators measuring disease burden at the collective and individual levels in each country. This study provides up-to-date information on whether ODA in the health sector is being provided to low- and middle-income countries based on disease burden. Although the selection of recipient countries remains a controversial topic, this research shows that ODA targeting has been performed well. Indeed, the global health community has been allocating DAH according to the level of disease burden.

## Figures and Tables

**Table 1:** Development assistance for health (DAH) to HIV/AIDS, tuberculosis, and malaria 2005–2017.

	2005	2006	2007	2008	2009	2010	2011	2012	2013	2014	2015	2016	2017
Global													
DAH	102,580	109,020	120,800	145,610	150,850	170,220	170,610	173,240	195,660	180,500	175,680	172,870	
Annual change (%)		6.3%	10.8%	20.5%	3.6%	12.8%	0.2%	1.5%	12.9%	−7.7%	−2.7%	−1.6%	
DAH per capita	2	2	2	2	2	2	2	2	3	2	2	2	
Annual change (%)		0%	0%	0%	0%	0%	0%	0%	50%	−33%	0%	0%	
DAH for HIV/AIDS	26,220	32,370	42,010	49,750	50,230	56,060	55,710	56,100	59,360	50,650	42,780	44,980	48,600
Annual change (%)		23.5%	29.8%	18.4%	1.0%	11.6%	−0.6%	0.7%	5.8%	−14.7%	−15.5%	5.1%	8.1%
DAH per capita for HIV/AIDS	1	1	1	2	2	2	2	2	2	2	1	1	2
Annual change (%)		0%	0%	100%	0%	0%	0%	0%	0%	0%	−50%	0%	100%
DAH for TB	5460	7160	9430	11,290	12,300	14,850	13,800	13,480	16,510	12,570	13,290	14,600	17,220
Annual change (%)		31.1%	31.7%	19.7%	8.9%	20.8%	−7.1%	−2.4%	22.5%	−23.8%	5.7%	9.9%	17.9%
DAH per capita for TB	0.285	0.302	0.320	0.330	0.348	0.348	0.345	0.341	0.349	0.333	0.333	0.327	0.341
Annual change (%)		5.9%	6.2%	2.8%	5.5%	0%	−0.9%	−1.1%	2.4%	−4.8%	0.2%	−1.8%	4.3%
DAH for malaria	7570	9190	9590	16,240	22,510	23,240	21,270	24,010	22,920	22,900	21,810	25,470	24,870
Annual change (%)		21.3%	4.4%	69.3%	38.6%	3.2%	−8.5%	12.9%	−4.5%	−0.1%	−4.8%	16.8%	−2.4%
DAH per capita for malaria	0.288	0.313	0.313	0.427	0.501	0.503	0.473	0.495	0.474	0.467	0.452	0.49	0.487
Annual change (%)		8.7%	0%	36.4%	17.3%	0.4%	−6.0%	4.7%	−4.2%	−1.5%	−3.2%	8.4%	−0.6%

Annual trend of Global Burden of Disease Health Financing are in constant (million of 2019 USD), DAH per capita (2019 USD), DAH = development assistance for heath.

**Table 2:** Associations between development assistance for health and disease burden (in 2005 and 2017) *.

Model	Regression Coefficient	95% CI	*p*	R^2^
HIV/AIDs model				
ln (DAH to DALY, 2017)	0.78	0.66, 0.91	<0.001	0.73
ln (DAH to DALY, 2005)	0.47	0.36, 0.58	<0.001	0.56
ln (DAH to number of infected people, 2017)	0.78	0.68, 0.88	<0.001	0.79
ln (DAH to number of infected people, 2005)	0.51	0.40, 0.61	<0.001	0.61
ln (DAH to number of deaths, 2017)	0.67	0.52, 0.81	<0.001	0.61
ln (DAH to number of deaths, 2005)	0.48	0.37, 0.61	<0.001	0.56
ln (DAH per capita to prevalence, 2017)	24.34	20.07, 28.61	<0.001	0.62
ln (DAH per capita to prevalence, 2005)	16.22	11.93, 20.51	<0.001	0.41
ln (DAH per capita to incidence rate, 2017)	1.19	1.02, 1.37	<0.001	0.69
ln (DAH per capita to incidence rate, 2005)	0.61	0.46, 0.76	<0.001	0.44
ln (DAH per capita to mortality rate, 2017)	0.42	0.31, 0.52	<0.001	0.46
ln (DAH per capita to mortality rate, 2005)	0.25	0.19, 0.32	<0.001	0.41
TB model				
ln (DAH to DALY, 2017)	0.66	0.46, 0.86	<0.001	0.46
ln (DAH to DALY, 2005)	0.78	0.55, 1.01	<0.001	0.49
ln (DAH to number of infected people, 2017)	0.67	0.44, 0.91	<0.001	0.42
ln (DAH to number of infected people, 2005)	0.51	0.27, 0.76	<0.001	0.34
ln (DAH to number of deaths, 2017)	0.66	0.45, 0.87	<0.001	0.45
ln (DAH to number of deaths, 2005)	0.58	0.34, 0.81	<0.001	0.38
ln (DAH per capita to prevalence, 2017)	203.93	137.07, 270.79	<0.001	0.33
ln (DAH per capita to prevalence, 2005)	7.28	−17.30, 31.88	0.55	0.05
ln (DAH per capita to incidence rate, 2017)	0.21	0.12, 0.31	<0.001	0.25
ln (DAH per capita to incidence rate, 2005)	−0.01	−0.03, 0.02	0.76	0.05
ln (DAH per capita to mortality rate, 2017)	0.19	0.11, 0.29	<0.001	0.21
ln (DAH per capita to mortality rate, 2005)	0.0008	−0.03, 0.03	0.96	0.05
Malaria model				
ln (DAH to DALY, 2017)	0.37	0.18, 0.55	<0.001	0.48
ln (DAH to DALY, 2005)	0.31	0.13, 0.49	0.001	0.41
ln (DAH to number of infected people, 2017)	0.33	0.18, 0.49	<0.001	0.49
ln (DAH to number of infected people, 2005)	0.28	0.13, 0.44	<0.001	0.42
ln (DAH to number of deaths, 2017)	0.39	0.19, 0.61	<0.001	0.47
ln (DAH to number of deaths, 2005)	0.22	0.05, 0.38	<0.001	0.36
ln (DAH per capita to prevalence, 2017)	0.84	−0.48, 2.16	0.208	0.33
ln (DAH per capita to prevalence, 2005)	0.77	−0.01, 1.55	0.05	0.11
ln (DAH per capita to incidence rate, 2017)	0.08	0.01, 0.15	0.02	0.37
ln (DAH per capita to incidence rate, 2005)	0.09	0.03, 0.15	<0.01	0.18
ln (DAH per capita to mortality rate, 2017)	0.03	−0.05, 0.12	0.45	0.32
ln (DAH per capita to mortality rate, 2005)	0.02	−0.04, 0.08	0.52	0.05

* Dependent variable: DAH, independent variable: DALY, covariate: GNI per capita. We used the total amount of DAHs and DAH per capita as a dependent variable. Disease burden was used for the independent variables. Number of infected people and the total number of deaths were used for the disease burden as an independent variable when the dependent variable was the total amount of DAH. Incidence, prevalence, and mortality were used when the dependent variable was DAH per capita.

**Table 3:** Trend of development assistance for health targeting in 2005–2017 (slope changes in regression line, results of likelihood ratio tests, fixed effects model) *.

Model	LR Test	*p*
HIV/AIDS		
fixed effects model: year × HIV DALY	47.26	<0.001
fixed effects model: year × HIV number of infected people	45.70	<0.001
fixed effects model: year × HIV number of deaths	50.88	<0.001
fixed effects model: year × HIV prevalence	15.77	<0.001
fixed effects model: year × HIV incidence rate	7.83	<0.001
fixed effects model: year × HIV mortality rate	17.69	<0.001
Malaria		
fixed effects model: year × malaria DALY	39.73	<0.001
fixed effects model: year ×malaria number of infected people	38.91	<0.001
fixed effects model: year× malaria number of deaths	42.22	<0.001
fixed effects model: year × malaria prevalence	56.69	<0.001
fixed effects model: year × malaria incidence rate	60.44	<0.001
fixed effects model: year × malaria mortality rate	55.87	<0.001
(Tuberculosis) TB		
fixed effects model: year × TB DALY	14.16	<0.001
fixed effects model: year × TB number of infected people	14.26	<0.001
fixed effects model: year × TB number of deaths	14.92	<0.001
fixed effects model: year × TB prevalence	16.45	<0.001
fixed effects model: year × TB incidence rate	16.92	<0.001
fixed effects model: year × TB mortality rate	18.00	<0.001

* Dependent variable: DAH, independent variable: DALY, covariate: GNI per capita. We allowed the slope to vary year by year in one model, and assumed the same slope in another model over time, and then ran the likelihood ratio test between the two models.

## Data Availability

The data used in this study are publicly available on the WHO, IHME, World Bank online databases.

## Supplementary Materials

The following supporting information can be downloaded at: https://www.mdpi.com/article/10.3390/ijerph192114091/s1, Table S1. Sources of data included in main regression analysis. Table S2. Countries eligible for inclusion and utilized in each regression analysis. Table S3. Descriptive statistics for HIV/AIDS panel sample of 92 countries, 2005–2017. Table S3.1. Descriptive statistics for log (HIV/AIDS) panel sample of 92 countries, 2005-2017. Table S4. Descriptive statistics for Tuberculosis panel sample of 87 countries, 2005–2017. Table S4.1 Descriptive statistics for log (Tuberculosis) panel sample of 87 countries, 2005–2017. Table S5. Descriptive statistics for Malaria panel sample of 75 countries, 2005–2017. Table S5.1. Descriptive statistics for log (Malaria) panel sample of 75 countries, 2005–2017. Table S6. DAH total amount and number of DALY people or number of cases by year (All countries). Table S7. DAH total amount and number of infected people or number of cases by year (All countries). Table S8. DAH total amount and number of deaths people or number of cases by year (All countries). Table S9. DAH per capita amount and number of prevalence people or number of cases by year (All countries). Table S10. DAH per capita amount and number of incidence people or number of cases by year (All countries). Table S11. DAH per capita amount and number of mortality people or number of cases by year (All countries).
