# Supplementary material for: Association between Development Assistance for Health and Disease Burden: A Longitudinal Analysis on Official Development Assistance for HIV/AIDS, Tuberculosis, and Malaria in 2005–2017
Source: Int J Environ Res Public Health. 2022 Oct 28;19(21):14091. doi: 10.3390/ijerph192114091 (PMC9656851; doi:10.3390/ijerph192114091)
Supplement: Supplementary file 1 [file ijerph-19-14091-s001.zip › ijerph-1957182-supplementary.pdf]

Supplementary material

Association between development assistance for health and disease burden: a longitudinal analysis on official development assistance for HIV/AIDS, tuberculosis and malaria in 2005-2017

Supp. Table S1. Sources of data included in main regression analysis.

Variable	Source
Development Assistance for Health by targeted disease	Institute for Health Metrics and Evaluation
HIV/AIDs, TB, Malaria DALY	Institute for Health Metrics and Evaluation
HIV/AIDs, TB, Malaria Incidence	World Health Organization
HIV/AIDs, TB, Malaria Death	Institute for Health Metrics and Evaluation
HIV/AIDs, TB, Malaria Infectious	World Health Organization
Population	World Bank Development Indicator

Supp. Table S2. Countries eligible for inclusion and utilized in each regression analysis.

HIV/AIDs (country code)	TB (country code)	Malaria (country code)
Afghanistan (AF)	Afghanistan (AF)	
	Albania (AL)	
		Algeria(DZ)
Angola (AO)	Angola(AO)	Angola(AO)
Armenia (AM)	Armenia(AM)	

	Azerbaijan(AZ)	
		Argentina(AR)
Bangladesh (BD)	Bangladesh(BD)	Bangladesh(BD)
Belarus (BY)	Belarus(BY)	
Benin (BJ)		Benin(BJ)
Bolivia (BO)	Bolivia(BO)	Bolivia(BO)
Bosnia and Herzegovina (BA)		
Botswana (BW)	Botswana(BW)	Botswana(BW)
Brazil (BR)	Brazil(BR)	Brazil(BR)
Burkina Faso (BF)	Burkina Faso(BF)	Burkina Faso(BF)
Burundi (BI)	Burundi(BI)	Burundi(BI)
Cambodia (KH)	Cambodia(KH)	Cambodia(KH)
Cameroon (CM)	Cameroon(CM)	Cameroon(CM)
Central African Republic (CF)		Central African Republic(CF)
Chad (TD)		Chad(TD)
China (People's Republic of) (CN)	China (People's Republic of) (CN)	China (People's Republic of) (CN)

Colombia (CO)		Colombia(CO)
Congo (CG)		Congo(CG)
Costa Rica (CR)		
Cote d'Ivoire (CI)	Cote d'Ivoire(CI)	Cote d'Ivoire(CI)
Cuba (CU)		
		Democratic People's Republic of Korea (KP)
Democratic Republic of the Congo (CD)	Democratic Republic of the Congo(CD)	Democratic Republic of the Congo(CD)
Djibouti (GI)	Djibouti(GI)	
Dominica (DM)		
Dominican Republic (DO)	Dominican Republic(DO)	Dominican Republic(DO)
Ecuador (EC)	Ecuador(EC)	
Egypt (EG)	Egypt(EG)	
El Salvador (SV)		
		Equatorial Guinea(GQ)
Eritrea (ER)	Eritrea(ER)	Eritrea(ER)
Ethiopia (ET)	Ethiopia(ET)	Ethiopia(ET)

Fiji (FJ)	Fiji(FJ)	
	Gabon(GA)	Gabon(GA)
	Gambia(GM)	
Georgia (GE)	Georgia(GE)	
Ghana (GH)	Ghana(GH)	Ghana(GH)
Guatemala (GT)		Guatemala(GT)
Guinea (GN)	Guinea(GN)	Guinea(GN)
Guinea-Bissau (GW)	Guinea-Bissau(GW)	Guinea-Bissau(GW)
Guyana (GY)		
Haiti (HT)	Haiti(HT)	Haiti(HT)
Honduras (HN)	Honduras(HN)	
India (IN)	India(IN)	India(IN)
Indonesia (ID)	Indonesia(ID)	Indonesia(ID)
		Iran(IR)
Iraq (IQ)	Iraq(IQ)	
	Jordan(JO)	

Jamaica (JM)		
	Kazakhstan(KZ)	
Kenya (KE)	Kenya(KE)	Kenya(KE)
Kiribati (KI)	Kiribati(KI)	
Kyrgyzstan (KG)	Kyrgyzstan(KG)	
Lebanon (LB)		
Lesotho (LS)	Lesotho(LS)	
Liberia (LR)	Liberia(LR)	Liberia(LR)
Libya (LY)	Libya(LY)	
Madagascar (MG)	Madagascar(MG)	Madagascar(MG)
Malawi (MW)	Malawi(MW)	Malawi(MW)
Malaysia (MY)	Malaysia(MY)	Malaysia(MY)
Mali (ML)		Mali(ML)
	Marshall Islands(MH)	
	Mauritania(MR)	Mauritania(MR)
	Mexico(MX)	

Mauritius (MU)		
Mongolia (MN)	Mongolia(MN)	
	Montenegro(ME)	
	Morocco(MA)	
Mozambique (MZ)	Mozambique(MZ)	Mozambique(MZ)
Myanmar (MM)	Myanmar(MM)	Myanmar(MM)
Namibia (NA)	Namibia(NA)	
Nepal (NP)	Nepal(NP)	Nepal(NP)
Nicaragua (NI)	Nicaragua(NI)	
Niger (NE)	Niger(NE)	Niger(NE)
Nigeria (NG)	Nigeria(NG)	Nigeria(NG)
Pakistan (PK)	Pakistan(PK)	
Papua New Guinea (PG)	Papua New Guinea(PG)	Papua New Guinea(PG)
	Paraguay(PY)	
Peru (PE)	Peru(PE)	Peru(PE)
Philippines (PH)	Philippines(PH)	Philippines(PH)

Rwanda (RW)	Rwanda(RW)	Rwanda(RW)
Saint Vincent and the Grenadines (VC)		
	Sao Tome and Principe(ST)	Sao Tome and Principe(ST)
Senegal (SN)	Senegal(SN)	Senegal(SN)
Serbia (RS)	Serbia(RS)	
Sierra Leone (SL)	Sierra Leone(SL)	Sierra Leone(SL)
	Solomon Islands(SB)	Solomon Islands(SB)
Somalia (SO)	Somalia(SO)	Somalia(SO)
South Africa (ZA)	South Africa(ZA)	South Africa(ZA)
Sri Lanka (LK)		Sri Lanka(LK)
	South Sudan(SS)	South Sudan(SS)
Sudan (SD)	Sudan(SD)	Sudan(SD)
Suriname (SR)		
Tanzania (TZ)		Tanzania(TZ)
	Tajikistan(TJ)	
Thailand (TH)	Thailand(TH)	Thailand(TH)

Timor-Leste (TL)	Timor-Leste(TL)	Timor-Leste(TL)
Togo (TG)		Togo(TG)
Tonga (TO)		
	Tunisia(TN)	
	Turkey(TR)	
Turkmenistan (TM)	Turkmenistan(TM)	
Uganda (UG)	Uganda(UG)	Uganda(UG)
Ukraine (UA)	Ukraine(UA)	
Uzbekistan (UZ)	Uzbekistan(UZ)	
Vanuatu (VU)		Vanuatu(VU)
		Venezuela(VE)
Viet Nam (VN)	Viet Nam(VN)	Viet Nam(VN)
Yemen (YE)	Yemen(YE)	
Zambia (ZM)	Zambia(ZM)	Zambia(ZM)
Zimbabwe (ZW)	Zimbabwe(ZW)	Zimbabwe(ZW)

Supp.Table S3. Descriptive statistics for HIV/AIDS panel sample of 92 countries, 2005-2017

Variable	Obs	Mean	Std. Dev.	Min	Max
Total DAH	1196	49507.174	95426.763	0	611053
GNI per capita	1165	2810.318	2551.531	140	12790
DAH per capita	1196	3.524	7.534	0	93
DALY	1183	890437.23	2195938.3	29.701	17128947
Number of infected people	1196	317025.64	780096.09	6.768	6683339.8
Number of deaths	1196	15675.084	39580.247	0	320241.44
Prevalence	1183	.015	.029	0	.165
Incidence	1183	1.651	3.462	0	24.1
Mortality	1183	69.856	132.589	0	852.686

Supp. Table S3.1. Descriptive statistics for log(HIV/AIDS) panel sample of 92 countries, 2005-2017

Variable	Obs	Mean	Std. Dev.	Min	Max
log(Total DAH)	1196	9.119	2.235	0	13.323
log (GNI per capita)	1165	7.491	1.004	4.949	9.456
log (DAH per capita)	1196	.949	.927	0	4.543
log (DALY)	1183	11.266	2.841	3.424	16.656
log (Number of infected people)	1196	10.309	2.832	2.05	15.715

log (Number of deaths)	1196	7.277	2.756	0	12.677
log (Prevalence)	1183	.014	.028	0	.152
log (Incidence)	1183	.607	.712	0	3.223
log (Mortality)	1183	2.879	1.758	0	6.75

Supp Table S4. Descriptive statistics for Tuberculosis panel sample of 87 countries, 2005-2017

Variable	Obs	Mean	Std. Dev.	Min	Max
Total DAH	1131	7291.736	13734.495	0	186890.56
GNI per capita	1093	2816.697	2657.668	140	12790
DAH per capita	1131	.474	.851	0	11.652
DALY	1131	550525.43	2025266.7	143.216	20432780
Number of infected people	1131	83027.692	307689.22	0	2901993.5
Number of deaths	1131	13402.016	53297.438	0	520807.32
Prevalence	1131	.002	.002	0	.008
Incidence	1131	221.266	207.561	0	1280
Mortality	1131	29.328	30.25	0	173.431

Supp. Table S4.1. Descriptive statistics for log(Tuberculosis) panel sample of 87 countries, 2005-2017

Variable	Obs	Mean	Std. Dev.	Min	Max
log(Total DAH)	1131	7.333	2.552	0	12.138
log (GNI per capita)	1093	7.491	.995	4.949	9.456
log (DAH per capita)	1131	.307	.352	0	2.538
log (DALY)	1131	11.203	2.257	4.971	16.833

log (Number of infected people)	1131	9.599	1.983	0	14.881
log (Number of deaths)	1131	7.486	2.196	0	13.163
log (Prevalence)	1131	.002	.002	0	.008
log (Incidence)	1131	4.914	1.153	0	7.155
log (Mortality)	1131	2.857	1.162	0	5.162

Supp. Table S5. Descriptive statistics for Malaria panel sample of 75 countries, 2005-2017

Variable	Obs	Mean	Std. Dev.	Min	Max
Total DAH	884	17743.958	29693.625	0	294178
GNI per capita	844	2503.685	2838.043	140	14250
DAH per capita	884	1.233	2.511	0	29.303
DALY	884	845043.84	2264558.9	0	19138730
Number of infected people	884	3389131.8	7933275.8	0	60694992
Number of deaths	884	11226.931	28960.616	0	241186.1
Prevalence	884	.144	.16	0	.596
Incidence	874	148.51	164.734	0	613.79
Mortality	884	44.36	62.274	0	283.015

Supp. Table S5.1. Descriptive statistics for log(Malaria) panel sample of 75 countries, 2005-2017

Variable	Obs	Mean	Std. Dev.	Min	Max
log(Total DAH)	884	8.134	2.572	0	12.592

log (GNI per capita)	844	7.283	1.037	4.949	9.565
log (DAH per capita)	884	.552	.609	0	3.411
log (DALY)	884	10.755	3.369	0	16.767
log (Number of infected people)	884	12.65	3.258	0	17.921
log (Number of deaths)	884	6.457	3.35	0	12.393
log (Prevalence)	884	.126	.134	0	.468
log (Incidence)	874	3.642	2.134	0	6.421
log (Mortality)	884	2.349	1.978	0	5.649

Supp. Table S6. DAH total amount and number of DALY people or number of cases by year (All countries)

	HIV/AIDS				TB				Malaria			
**year**	**Coef.**	**95%CI**	**p**	**R**	**Coef.**	**95%CI**	**p-value**	**R**	**Coef.**	**95%CI**	**p-value**	**R**
**2005**	0.47	0.36, 0.58	<0.001	0.56	0.78	0.55, 1.01	<0.001	0.49	0.31	0.13, 0.49	0.001	0.41
**2006**	0.52	0.41, 0.63	<0.001	0.61	0.69	0.48, 0.91	<0.001	0.47	0.26	0.06, 0.47	0.01	0.28
**2007**	0.54	0.44, 0.64	<0.001	0.65	0.62	0.42, 0.83	<0.001	0.40	0.25	0.08, 0.41	<0.01	0.37
**2008**	0.48	0.38 ,0.59	<0.001	0.60	0.56	0.38, 0.75	<0.001	0.41	0.25	0.08, 0.42	<0.01	0.43
**2009**	0.48	0.37, 0.59	<0.001	0.58	0.54	0.33, 0.76	<0.001	0.34	0.33	0.12, 0.53	<0.01	0.35
**2010**	0.51	0.39, 0.62	<0.001	0.59	0.52	0.32, 0.73	<0.001	0.35	0.33	0.16, 0.51	<0.001	0.46
**2011**	0.56	0.45, 0.66	<0.001	0.65	0.68	0.46, 0.90	<0.001	0.36	0.39	0.18, 0.59	<0.001	0.41
**2012**	0.56	0.46 ,0.67	<0.001	0.66	0.53	0.33, 0.74	<0.001	0.36	0.44	0.21, 0.67	<0.001	0.37
**2013**	0.58	0.46, 0.69	<0.001	0.63	0.69	0.49, 0.89	<0.001	0.47	0.23	0.07, 0.40	<0.01	0.45
**2014**	0.63	0.51, 0.75	<0.001	0.67	0.63	0.43, 0.82	<0.001	0.45	0.31	0.14, 0.47	<0.001	0.51
**2015**	0.63	0.50, 0.76	<0.001	0.59	0.65	0.44, 0.86	<0.001	0.45	0.42	0.24, 0.61	<0.001	0.45
**2016**	0.71	0.57, 0.82	<0.001	0.67	0.61	0.43, 0.78	<0.001	0.48	0.37	0.19, 0.55	<0.001	0.48

**2017**	0.78	0.66, 0.91	<0.001	0.73	0.66	0.46, 0.86	<0.001	0.46	0.37	0.18, 0.55	<0.001	0.48

Supp.Table S7. DAH total amount and number of infected people or number of cases by year (All countries)

	**HIV/AIDS**				**TB**				**Malaria**			
**year**	**Coef.**	**95%CI**	**p**	**R**	**Coef.**	**95%CI**	**p-value**	**R**	**Coef.**	**95%CI**	**p-value**	**R**
**2005**	0.51	0.40, 0.61	<0.001	0.61	0.51	0.27, 0.76	<0.001	0.34	0.28	0.13, 0.44	<0.001	0.42
**2006**	0.55	0.45, 0.64	<0.001	0.65	0.71	0.46, 0.96	<0.001	0.44	0.50	0.30, 0.707	<0.001	0.43
**2007**	0.57	0.47, 0.66	<0.001	0.68	0.65	0.42, 0.89	<0.001	0.37	0.45	0.30, 0.61	<0.001	0.54
**2008**	0.50	0.40, 0.60	<0.001	0.63	0.60	0.40, 0.81	<0.001	0.40	0.40	0.24, 0.57	<0.001	0.53
**2009**	0.49	0.39, 0.60	<0.001	0.59	0.57	0.33, 0.81	<0.001	0.32	0.54	0.35, 0.72	<0.001	0.51
**2010**	0.52	0.41, 0.63	<0.001	0.62	0.53	0.30, 0.76	<0.001	0.31	0.45	0.29, 0.61	<0.001	0.56
**2011**	0.57	0.47, 0.66	<0.001	0.69	0.73	0.48, 0.98	<0.001	0.34	0.46	0.27, 0.66	<0.001	0.47
**2012**	0.57	0.48, 0.66	<0.001	0.69	0.54	0.30, 0.77	<0.001	0.32	0.48	0.26, 0.69	<0.001	0.41
**2013**	0.59	0.48, 0.69	<0.001	0.67	0.69	0.46, 0.93	<0.001	0.41	0.34	0.19, 0.49	<0.001	0.53
**2014**	0.63	0.53, 0.73	<0.001	0.71	0.66	0.44, 0.88	<0.001	0.42	0.39	0.25, 0.53	<0.001	0.59
**2015**	0.63	0.52, 0.75	<0.001	0.65	0.66	0.42, 0.90	<0.001	0.41	0.48	0.31, 0.64	<0.001	0.52
**2016**	0.70	0.60, 0.80	<0.001	0.74	0.65	0.45, 0.85	<0.001	0.46	0.42	0.26, 0.58	<0.001	0.55

**2017**	0.78	0.68 0.88	<0.001	0.79	0.67	0.44, 0.91	<0.001	0.42	0.33	0.18, 0.49	<0.001	0.49

Supp. Table S8. DAH total amount and number of deaths people or number of cases by year (All countries)

	**HIV/AIDS**				**TB**				**Malaria**			
**year**	**Coef.**	**95%CI**	**p**	**R**	**Coef.**	**95%CI**	**p-value**	**R**	**Coef.**	**95%CI**	**p-value**	**R**
**2005**	0.48	0.37, 0.61	<0.001	0.56	0.58	0.34, 0.81	<0.001	0.38	0.22	0.05, 0.38	0.009	0.36
**2006**	0.53	0.43, 0.64	<0.001	0.61	0.69	0.46, 0.91	<0.001	0.46	0.24	0.04, 0.44	0.01	0.28
**2007**	0.55	0.45, 0.66	<0.001	0.63	0.62	0.40, 0.83	<0.001	0.38	0.22	0.06, 0.38	0.006	0.37
**2008**	0.50	0.39, 0.60	<0.001	0.61	0.56	0.36, 0.75	<0.001	0.39	0.23	0.07, 0.40	0.005	0.42
**2009**	0.50	0.38, 0.61	<0.001	0.57	0.52	0.30, 0.75	<0.001	0.31	0.31	0.11, 0.50	0.002	0.34
**2010**	0.52	0.40, 0.64	<0.001	0.59	0.51	0.30, 0.73	<0.001	0.33	0.30	0.12, 0.49	0.001	0.43
**2011**	0.58	0.47, 0.68	<0.001	0.65	0.68	0.44, 0.91	<0.001	0.34	0.35	0.14, 0.56	0.002	0.39
**2012**	0.59	0.48, 0.69	<0.001	0.66	0.52	0.30, 0.73	<0.001	0.34	0.43	0.19, 0.19	0.001	0.36
**2013**	0.61	0.49, 0.72	<0.001	0.63	0.68	0.47, 0.90	<0.001	0.44	0.22	0.04, 0.39	0.01	0.44
**2014**	0.66	0.54, 0.78	<0.001	0.67	0.63	0.43, 0.84	<0.001	0.44	0.27	0.10, 0.44	0.002	0.48
**2015**	0.65	0.52, 0.79	<0.001	0.59	0.64	0.42, 0.86	<0.001	0.43	0.39	0.20, 0.58	<0.001	0.41

2016	0.72	0.59, 0.85	<0.001	0.67	0.62	0.44, 0.80	<0.001	0.47	0.36	0.17, 0.55	<0.001	0.46
**2017**	0.67	0.52, 0.81	<0.001	0.61	0.66	0.45, 0.87	<0.001	0.45	0.39	0.19, 0.61	<0.001	0.47

Supp. Table S9. DAH per capita amount and number of prevalence people or number of cases by year (All countries)

	**HIV/AIDS**				**TB**				**Malaria**			
**year**	**Coef.**	**95%CI**	**p**	**R**	**Coef.**	**95%CI**	**p-value**	**R**	**Coef.**	**95%CI**	**p-value**	**R**
**2005**	16.22	11.93, 20.51	<0.001	0.41	7.28	-17.30, 31.88	0.55	0.05	0.77	-0.01, 1.55	0.05	0.11
**2006**	18.86	14.04, 23.68	<0.001	0.42	57.44	4.92, 109.95	0.03	0.05	0.91	-0.09, 1.92	0.07	0.05
**2007**	21.83	17.05, 26.61	<0.001	0.50	62.82	11.48, 114.17	0.01	0.06	0.48	-0.25, 1.22	0.19	0.08
**2008**	24.34	18.76, 29.92	<0.001	0.48	60.79	16.50, 105.08	<0.001	0.08	0.44	-0.72, 1.62	0.44	0.11
**2009**	24.54	19.14, 29.94	<0.001	0.49	53.92	-5.67, 113.53	0.07	0.03	0.84	-0.50, 2.20	0.21	0.09
**2010**	24.68	19.29, 30.07	<0.001	0.50	27.59	-36.20, 91.40	0.39	0.01	0.72	-0.57, 2.02	0.27	0.20
**2011**	24.91	19.67, 30.15	<0.001	0.51	10.43	-38.46, 59.32	0.67	0.002	0.78	-0.28, 1.84	0.14	0.28
**2012**	25.58	20.29, 30.87	<0.001	0.53	59.15	-15.67, 133.97	0.12	0.04	1.20	-0.14, 2.55	0.07	0.15
**2013**	24.35	19.28, 29.43	<0.001	0.53	38.63	-24.43, 101.71	0.22	0.05	0.36	-1.02, 1.75	0.60	0.22
**2014**	24.52	19.59, 29.45	<0.001	0.56	47.76	-7.51, 103.04	0.08	0.04	0.59	-0.63, 1.82	0.33	0.25
**2015**	22.92	18.51, 27.32	<0.001	0.56	29.27	-32.31, 90.85	0.34	0.05	0.77	-0.281, 0.83	0.15	0.26

**2016**	24.24	20.14, 28.35	<0.001	0.63	149.98	90.47, 209.49	<0.001	0.24	0.88	-0.452, 0.22	0.19	0.30
**2017**	24.34	20.07, 28.61	<0.001	0.62	203.93	137.07, 270.79	<0.001	0.33	0.84	-0.48, 2.16	0.20	0.33

Supp. Table S10. DAH per capita amount and number of incidence people or number of cases by year (All countries)

	HIV/AIDS				TB				Malaria			
**year**	**Coef.**	**95%CI**	**p**	**R**	**Coef.**	**95%CI**	**p-value**	**R**	**Coef.**	**95%CI**	**p-value**	**R**
**2005**	0.61	0.46, .76	<0.001	0.44	-0.01	-0.03, 0.02	0.76	0.05	0.09	0.03, 0.15	<0.01	0.18
**2006**	0.71	0.53, .88	<0.001	0.43	0.08	0.02, 0.15	0.01	0.08	0.12	0.04, 0.19	<0.01	0.15
**2007**	0.83	0.65, 1.00	<0.001	0.52	0.07	0.01, 0.13	0.03	0.05	0.07	0.02, 0.13	<0.01	0.18
**2008**	0.87	0.66, 1.09	<0.001	0.44	0.08	0.03, 0.13	<0.01	0.11	0.10	0.02, 0.18	0.01	0.18
**2009**	0.89	0.67, 1.10	<0.001	0.45	0.09	0.02, 0.17	<0.01	0.08	0.16	0.07, 0.25	0.001	0.23
**2010**	0.95	0.75, 1.16	<0.001	0.51	0.05	-0.02, 0.12	0.20	0.02	0.13	0.04, 0.22	<0.01	0.29
**2011**	0.97	0.77, 1.18	<0.001	0.52	0.03	-0.03, 0.10	0.37	0.01	0.09	0.01, 0.16	0.01	0.31
**2012**	1.00	0.78, 1.22	<0.001	0.50	0.12	0.03, 0.21	<0.01	0.09	0.12	0.03, 0.21	<0.01	0.21
**2013**	1.02	0.81, 1.22	<0.001	0.54	0.10	0.02, 0.18	0.01	0.10	0.10	0.02, 0.19	0.01	0.29
**2014**	1.03	0.82, 1.24	<0.001	0.55	0.10	0.03, 0.17	<0.01	0.11	0.07	-0.002, 0.14	0.05	0.28

**2015**	1.00	0.82, 1.19	<0.001	0.57	0.09	0.016, 0.16	0.01	0.10	0.08	0.02, 0.14	<0.01	0.32
**2016**	1.12	0.95, 1.29	<0.001	0.68	0.18	0.10, 0.25	<0.001	0.23	0.09	0.02, 0.16	0.01	0.35
**2017**	1.19	1.02, 1.37	<0.001	0.69	0.21	0.12, 0.31	<0.001	0.25	0.08	0.01, 0.15	0.02	0.37

Supp. Table S11. DAH per capita amount and number of mortality people or number of cases by year (All countries)

HIV/AIDS					TB				Malaria			
**year**	**Coef.**	**95%CI**	**p**	**R**	**Coef.**	**95%CI**	**p-value**	**R**	**Coef.**	**95%CI**	**p-value**	**R**
**2005**	0.25	0.19, 0.32	<0.001	0.41	0.0008	-0.03, 0.03	0.96	0.05	0.02	-0.04, 0.08	0.52	0.05
**2006**	0.31	0.23, 0.39	<0.001	0.45	0.09	0.01, 0.17	0.01	0.07	0.03	-0.04, 0.11	0.42	0.01
**2007**	0.35	0.27, 0.43	<0.001	0.50	0.07	0.0008, 0.15	0.04	0.04	-0.0005	-0.05, 0.05	0.98	0.06
**2008**	0.38	0.28, 0.47	<0.001	0.43	0.06	-0.006, 0.12	0.07	0.03	-0.01	-0.10, 0.07	0.74	0.10
**2009**	0.37	0.28, 0.46	<0.001	0.43	0.05	-0.03, 0.14	0.20	0.01	0.02	-0.07, 0.13	0.57	0.07
**2010**	0.39	0.30, 0.49	<0.001	0.47	0.04	-0.04, 0.14	0.34	0.01	0.01	-0.08, 0.11	0.76	0.19
**2011**	0.40	0.31, 0.50	<0.001	0.47	0.005	-0.06, 0.07	0.88	0.0004	0.03	-0.04, 0.11	0.26	0.43
**2012**	0.42	0.32, 0.52	<0.001	0.45	0.08	-0.01, 0.18	0.10	0.04	0.03	-0.06, 0.14	0.44	0.12
**2013**	0.44	0.35, 0.53	<0.001	0.52	0.06	-0.01, 0.15	0.11	0.06	-0.01	-0.11, 0.08	0.77	0.22

**2014**	0.42	0.32, 0.52	<0.001	0.48	0.05	-0.01, 0.13	0.12	0.03	0.02	-0.06, 0.11	0.64	0.24
**2015**	0.41	0.32, 0.50	<0.001	0.49	0.06	-0.01, 0.14	0.11	0.07	0.05	-0.01, 0.13	0.14	0.26
**2016**	0.46	0.37, 0.55	<0.001	0.59	0.15	0.07, 0.23	<0.001	0.15	0.04	-0.05, 0.13	0.41	0.29
**2017**	0.42	0.31, 0.52	<0.001	0.46	0.19	0.11, 0.29	<0.001	0.21	0.03	-0.05, 0.12	0.45	0.32
